# Investigation of the added value of CT-based radiomics in predicting the development of brain metastases in patients with radically treated stage III NSCLC

**DOI:** 10.1177/17588359221116605

**Published:** 2022-08-22

**Authors:** Simon A. Keek, Esma Kayan, Avishek Chatterjee, José S. A. Belderbos, Gerben Bootsma, Ben van den Borne, Anne-Marie C. Dingemans, Hester A. Gietema, Harry J. M. Groen, Judith Herder, Cordula Pitz, John Praag, Dirk De Ruysscher, Janna Schoenmaekers, Hans J. M. Smit, Jos Stigt, Marcel Westenend, Haiyan Zeng, Henry C. Woodruff, Philippe Lambin, Lizza Hendriks

**Affiliations:** The D-Lab, Department of Precision Medicine, GROW – School for Oncology and Reproduction, Maastricht University, Maastricht, The Netherlands; The D-Lab, Department of Precision Medicine, GROW – School for Oncology and Reproduction, Maastricht University, Maastricht, The Netherlands; The D-Lab, Department of Precision Medicine, GROW – School for Oncology and Reproduction, Maastricht University, Maastricht, The Netherlands; Department of Radiation Oncology, The Netherlands Cancer Institute, Amsterdam, The Netherlands; Department of Pulmonary Diseases, Zuyderland Hospital, Heerlen, The Netherlands; Department of Pulmonary Diseases, Catharina Hospital, Eindhoven, The Netherlands; Department of Pulmonary Diseases, Erasmus MC, Rotterdam, The Netherlands; Department of Radiology and Nuclear Medicine, GROW – School for Oncology and Reproduction, Maastricht University Medical Centre+, Maastricht, The Netherlands; Department of Pulmonary Diseases, University Medical Center Groningen, University of Groningen, Groningen, The Netherlands; Department of Pulmonary Diseases, Meander Medical Center, Amersfoort, The Netherlands; Department of Pulmonary Diseases, Laurentius Hospital, Roermond, The Netherlands; Department of Radiotherapy, Erasmus MC, Rotterdam, The Netherlands; Department of Radiation Oncology (Maastro), GROW – School for Oncology and Reproduction, Maastricht University Medical Centre+, Maastricht, The Netherlands; Department of Pulmonary Diseases, GROW – School for Oncology and Reproduction, Maastricht University Medical Center+, Maastricht, The Netherlands; Department of Pulmonary Diseases, Rijnstate, Arnhem, The Netherlands; Department of Pulmonary Diseases, Isala Hospital, Zwolle, The Netherlands; Department of Pulmonary Diseases, VieCuri, Venlo, The Netherlands; Department of Radiation Oncology (Maastro), GROW – School for Oncology and Reproduction, Maastricht University Medical Centre+, Maastricht, The Netherlands; The D-Lab, Department of Precision Medicine, GROW – School for Oncology and Reproduction, Maastricht University, Maastricht, The Netherlands; Department of Radiology and Nuclear Medicine, GROW – School for Oncology, Maastricht University Medical Centre+, Maastricht, The Netherlands; The D-Lab, Department of Precision Medicine, GROW – School for Oncology and Reproduction, Maastricht University, Maastricht, The Netherlands; Department of Radiology and Nuclear Medicine, GROW – School for Oncology, Maastricht University Medical Centre+, Maastricht, The Netherlands; Department of Pulmonary Diseases, GROW – School for Oncology and Reproduction, Maastricht University Medical Centre+, P.O. Box 5800, 6202 AZ, Maastricht, The Netherlands

**Keywords:** CT, metastatic brain tumours, non-small-cell lung cancer, predictive biomarker, tumour biology

## Abstract

**Introduction:**

Despite radical intent therapy for patients with stage III non-small-cell lung cancer (NSCLC), cumulative incidence of brain metastases (BM) reaches 30%. Current risk stratification methods fail to accurately identify these patients. As radiomics features have been shown to have predictive value, this study aims to develop a model combining clinical risk factors with radiomics features for BM development in patients with radically treated stage III NSCLC.

**Methods:**

Retrospective analysis of two prospective multicentre studies. Inclusion criteria: adequately staged [^18^F-fluorodeoxyglucose positron emission tomography-computed tomography (18-FDG-PET-CT), contrast-enhanced chest CT, contrast-enhanced brain magnetic resonance imaging/CT] and radically treated stage III NSCLC, exclusion criteria: second primary within 2 years of NSCLC diagnosis and prior prophylactic cranial irradiation. Primary endpoint was BM development any time during follow-up (FU). CT-based radiomics features (*N* = 530) were extracted from the primary lung tumour on 18-FDG-PET-CT images, and a list of clinical features (*N* = 8) was collected. Univariate feature selection based on the area under the curve (AUC) of the receiver operating characteristic was performed to identify relevant features. Generalized linear models were trained using the selected features, and multivariate predictive performance was assessed through the AUC.

**Results:**

In total, 219 patients were eligible for analysis. Median FU was 59.4 months for the training cohort and 67.3 months for the validation cohort; 21 (15%) and 17 (22%) patients developed BM in the training and validation cohort, respectively. Two relevant clinical features (age and adenocarcinoma histology) and four relevant radiomics features were identified as predictive. The clinical model yielded the highest AUC value of 0.71 (95% CI: 0.58–0.84), better than radiomics or a combination of clinical parameters and radiomics (both an AUC of 0.62, 95% CIs of 0.47–076 and 0.48–0.76, respectively).

**Conclusion:**

CT-based radiomics features of primary NSCLC in the current setup could not improve on a model based on clinical predictors (age and adenocarcinoma histology) of BM development in radically treated stage III NSCLC patients.

## Introduction

The brain is a frequent site of disease relapse in patients with non-small-cell lung cancer (NSCLC). Risk factors for brain metastases (BM) are advanced stage, adenocarcinoma histology, and younger age.^1–3^ For radically treated patients, locally advanced (stage III) NSCLC has the highest risk for BM, with a cumulative incidence of BM of approximately 30%.^4^ The majority of BM present within 2 years of diagnosis, despite brain imaging without BM during initial staging for NSCLC.^4^ Brain magnetic resonance imaging (MRI) is recommended in clinical guidelines [and if not possible, contrast-enhanced computed tomography (CECT)].^5–8^ The type of chemotherapy administered during chemoradiation therapy does not influence the incidence of BM.^2^ Curative treatment of (symptomatic) BM is seldom possible and for the overwhelming majority of patients overall survival (OS) is limited.^9 [Bibr bibr10-17588359221116605]^ Moreover, BM are associated with a devastating impact on Quality of Life (QoL).^10,11^ Therefore, strategies to prevent BM and to predict who is at risk for their development are necessary, especially taking into consideration that treatments that reduce the incidence of BM are possible.

Prophylactic cranial irradiation (PCI) has been shown to reduce the incidence of BM in patients with NSCLC with a relative risk of 0.33.^4^ PCI prolongs progression-free survival in stage III NSCLC, but not OS.^4^ Furthermore, PCI leads to neurocognitive impairment (mostly grade 1–2) in about 25–27% of patients.^12,13^ Ideally, only those patients with an a priori high risk of BM should undergo PCI and those with a low risk could avoid the risk of neurocognitive decline. An alternative approach to preventive treatment would be to closely monitor patients at high risk for BM through MRI surveillance, although there is no evidence that this improves outcome.^14^ Hence, identifying predictive biomarkers, and thereby stratifying patients at high *versus* low risk for BM development, is key to personalize follow-up (FU) and treatment.

Although clinical risk factors are identified as described above, it remains challenging to discriminate between patients at high and low risk of BM.^15,16^ Won *et al.*^17^ developed a prediction model using clinical and pathological risk factors, such as histology, pathological T- and N-stages, and smoking status to predict the probability of BM development after curative surgery in a large group of patients with NSCLC.17 This study used dedicated brain imaging (majority brain MRI, subset brain CECT) at baseline to verify that no BM were present. However, the model only had a moderate discriminative power in predicting BM development at 2 and 5 years [Harrell’s C-index (CI) of 0.670 and 0.674, respectively], and was verified only through internal validation, showing a clear need for more studies investigating BM prediction models.

Metastases develop through a ‘wiring’ of the primary tumour to spread to certain organs (‘seed and soil’ hypothesis).^18–20^ Therefore, analysis of the primary tumour could provide valuable feedback in identifying those patients at risk of developing BM. Indeed, molecular biomarkers, such as microRNAs expression patterns, were previously associated with BM development in patients with NSCLC.^21,22^ However, these markers were not investigated in a prospective predictive study. Furthermore, they require invasive biopsies, and small tumour biopsies disregard the heterogeneous nature of tumours.^23^ Therefore, an approach that takes the entirety of the tumour into account (i.e. the whole primary tumour and not only a small biopsy) is preferred.

Radiomics refers to the extraction of quantitative data from medical images using mathematical algorithms and finding correlations with biological or clinical outcomes *via* machine learning techniques.^24–26^ When radiomics is applied to oncology, radiological images [e.g. CT, MRI, or positron emission tomography (PET)] performed during routine clinical workflow can be used to non-invasively extract imaging features describing the tumour and patient phenotypes.^27^ These features can have significant diagnostic, prognostic, and predictive values, and hold the potential to assist clinical decision-making.^28^

Coroller *et al.*^29^ found that a model based on the primary tumour in locally advanced adenocarcinomas of the lung was predictive of distant metastases. However, this study tried to predict distant metastases in general, not BM specifically. Three other studies showed that CT-based radiomics models on primary lung tumours might have positive value to predict BM in patients with NSCLC.^30–32^ Models of clinical features and radiomics features were compared and combined, and in all three studies complementary value for the radiomics models were found. However, sample sizes were small (*N* = 85–124), no external validation was performed, not all patients were adequately staged according to guidelines,^5–8^ and patient groups included were heterogeneous (e.g. different disease stages), which may affect the reliability of the created models.

Therefore, the aim of the current study is to develop a prediction model for BM development (low *versus* high risk) in patients with adequately staged, radically treated stage III NSCLC, based on clinical patient characteristics only, and combined with CT-based radiomics analysis of the primary lung tumour. We hypothesize that a model based on CT-radiomics and clinical variables can assist medical professionals in the decision-making process, and facilitate precision medicine for the treatment of NSCLC.

## Materials and methods

### Study population

This was a post hoc analysis of two prospective, multicentre studies [NVALT-11, NCT01282437 (inclusion 2009–2015) and NL3335 (inclusion 2012–2017)] enrolling patients with stage III NSCLC (IASLC 7th edition). NCT01282437 (*N* = 175) was a multicentre randomized phase III study evaluating PCI *versus* no PCI in patients with radically treated stage III NSCLC. Primary endpoint was the development of symptomatic BM 24 months after randomization. Approximately half of these patients had baseline brain CECT, the remaining brain MRI. Only patients without baseline BM were eligible.^33^ NL3335 was a prospective multicentre observational study, evaluating whether performing a brain MRI after a negative dedicated CECT had additive value in the diagnosis of asymptomatic BM.^34^ One of the secondary endpoints was the development of BM after radical treatment for stage III NSCLC. For NL3335, patients with stage III NSCLC and an available ^18^F-fluorodeoxyglucose (^18^F-FDG)-PET-CT were screened, and only those with a dedicated brain CT (with contrast, arms at thorax level, correct field of view, and delayed imaging^35^) performed before or together with the ^18^F-FDG-PET-CT available, and followed by a brain MRI, were deemed eligible. For the current study, all patients who were staged with ^18^F-FDG-PET-CT and dedicated brain imaging (MRI and/or CECT), and treated with radical intent therapy (i.e. sequential or concurrent chemoradiation with/without surgery, or radical radiotherapy), were eligible. For both studies, additional eligibility criteria consisted of availability of baseline chest CECT (i.e. at diagnosis of stage III NSCLC), and a distinct primary tumour [primary tumour not detectable (Tx) or primary tumour not definable due to surrounding atelectasis were excluded]. Furthermore, all patients that received PCI or had a second primary within 2 years of NSCLC diagnosis were excluded.

The dataset was split into a training and a validation dataset. The patient data obtained from the NL3335 study from the hospitals in Heerlen (Zuyderland MC) and Maastricht (Maastricht UMC+) were assigned to the training dataset. This dataset was used to select relevant features and to train the model. To test the performance on data not yet seen by the model, a validation dataset was also defined comprising data from one of the centres participating in the NL3335 study (VieCuri Medisch Centrum) and from the NVALT-11 study.

### Patient characteristics

Baseline characteristics recorded in the two prospective studies and extracted for this analysis included age, gender, World Health Organization Performance Status (WHO PS), smoking status, pack years, tumour, node, metastasis stage (IASLC 7th edition, IIIA *versus* IIIB), histology, and FU data regarding BM development. The primary endpoint of this study was the development of BM (binary: yes/no), which was defined as disease progression to the brain assessed by MRI or CECT anytime during FU.

### Image acquisition

Pre-treatment diagnostic chest CT images were acquired with a Philips Gemini TF64 (Philips Medical Systems, Best, Netherlands), Siemens Somatom Force scanner (Siemens Healthineers, Erlangen, Germany), GE Discovery STE (GE Medical systems, Chicago, IL, USA), and Toshiba Aquilion (Toshiba, Tokyo, Japan). The scanning parameters were 80–140 kVp tube voltage, 37–462 mAs tube current, and 512 × 512 matrix. An overview of the imaging characteristics can be found in Supplemental Figure S1. CT images were obtained through the picture archiving and communication system in the Digital Imaging and Communications in Medicine format. For each patient, an ^18^F-FDG-PET-CT with a non-diagnostic low-dose CT for attenuation correction and diagnostic CECT were available. Generally, the injection of contrast induces noise in the images and hence in some radiomics features due to differences between patients in diffusion of the contrast agent. However, the CECT scan was finally chosen for the analysis, as several tumours were difficult to contour on the low-dose CT due to mediastinal invasion and undefined tumour borders. Furthermore, the lower spatial resolution of low-dose CT could lead to the loss of important radiomics information. The CECT scans were obtained with different imaging parameters (e.g. spatial resolution, slice thickness, reconstruction kernel) due to variation in acquisition protocols of hospitals and different scanners available. Therefore, imaging parameters that were the most common throughout all images were set as the standard imaging parameters, for example, 3 mm slice thickness, soft reconstruction kernel, which were used to select the appropriate CECT scan for each patient accordingly.

### Tumour segmentation

The region of interest (ROI), that is, the primary lung tumour, was manually delineated on the CT images using MIM Software Inc. (Version 6.9.4, Cleveland, OH, USA). ^18^F-FDG-PET-CT imaging was used alongside the CT image to locate the tumour, and to identify tumour borders adjacent to atelectasis or tumours invading extrapulmonary structures. The lung window was used to identify tumour-lung borders, while tumour regions adjacent to extrapulmonary tissues were contoured in the mediastinal window. In cases of tumours completely (or for a greater part) surrounded by atelectasis (i.e. reliable contouring not possible), the CT scan was excluded from radiomics analysis. All tumour segmentations were performed and checked for accurate delineation by an experienced pulmonary oncologist or thoracic radiologist.

### Pre-processing and feature extraction

To homogenize the datasets prior to feature extraction, all images were resampled to the mode of the unprocessed scans (1 × 1 mm^2^ pixel size and 3 mm slice thickness). Furthermore, to reduce noise and computational burden, the intensity values inside the ROI were discretized with a fixed bin width of 25 Hounsfield units which has been reported to yield the most reproducible radiomics features for CT images.^36^

Feature extraction for every 3D ROI on each baseline CECT was performed using PyRadiomics version 2.2.0 on both the original images and filtered images. Laplacian of Gaussian (LoG) convolution filtering was applied to the original image to highlight the regions of intensity change within an image. The LoG was applied with five different Gaussian standard deviation (SD) values ranging from 1 to 5 mm resulting in five different LoG images. The radiomics features extracted from the images can be divided into three main groups: first-order intensity and histogram statistics features, shape and size features, and texture features. First-order intensity and histogram statistics features describe the voxel intensity distribution within the ROI. Shape and size features describe the spatial characteristics of the ROI itself, such as volume and sphericity, and are thus independent of the image contents. Texture features describe the spatial relationships of voxel intensities and are derived from six different matrices that are defined over the ROIs: grey-level co-occurrence (GLCM), grey-level run length, grey-level size zone (GLSZM), grey-level distance zone, neighbourhood grey-level dependence, and neighbourhood grey-tone difference matrix.

The total number of features that can be extracted with the PyRadiomics package, without using highly correlating/depreciated features and without any further manipulation of the image is 107. However, the application of image filters, either Wavelet based or Log based with different kernel sizes can multiply this number to thousands of features. The wavelet-based features were omitted from this analysis, as with a relatively low number of patients adding more features would increase the risk of overfitting and finding spurious correlations, and because wavelet-based features have shown to have low reproducibility compared to Log-filtered images.^37^

### Feature selection and predictive modelling

The radiomics features were first normalized on the training dataset through z-score normalization: the mean and SD of each feature were determined over the entire training population and used to perform normalization on the training dataset, as well as on the validation dataset. For the clinical features, a list of known clinical predictors for BM defined by Won *et al*. were used.^17^ These included histology (adenocarcinoma *versus* others), age, stage (IIIA *versus* IIIB), WHO PS (0 *versus* 1 or higher, 0–1 *versus* 2 or higher, and 0–2 *versus* 3), smoking status (ever *versus* never, and current *versus* former or ever), packyears, and treatment received (concurrent chemoradiation *versus* other). As the volume of the tumour is also a radiomics feature, it was not included as a clinical variable. Dimensionality reduction through feature selection was performed on both the radiomics and clinical variables.

Feature selection and modelling were performed using R software (Version 3.3.2, R Core Team, Vienna, Austria) on the training dataset.^38^ Supervised univariate feature selection was performed on all clinical and radiomics features, using the occurrence of BM as the binary outcome. For each feature, the area under the curve (AUC) of the receiving operating characteristic (ROC) was calculated. The ROC curve shows the sensitivity and specificity of the model at different classification thresholds on the feature score. The AUC of this curve was a metric of the predictive performance of the feature, ranging from 0.5 to 1, where 1 indicates a perfect prediction and 0.5 a prediction equal to chance. As an AUC > 0.6 indicates a feature has some predictive power, this cut-off was chosen to select features. Features that are highly correlated (Spearman’s correlation > 0.8) were determined, and the feature with the highest average correlation with all other features remaining in the set was excluded. To verify that radiomics features are not simply surrogates for tumour volume, the correlation with volume was also determined. Three separate models were created: using the selected radiomics features, using the selected clinical features, and using a combination of selected radiomics and clinical features.

Using the selected features, a generalized linear model was trained on the training dataset using BM status as outcome calculated. Without changing its parameters, the model was then validated on the validation dataset, and the prediction score created as output. This prediction score is the probability a patient will develop a BM, and ranges from 0 to 1. By selecting a threshold on this prediction score, the binary classification of the validation patients was performed.

### Statistical analysis

Baseline patient characteristics were analysed using standard descriptive statistics. Statistical analysis of continuous variables was performed with the independent two-sample *t*-test, whereas differences in categorical variables were analysed using a χ^2^-test. The reported statistical significance levels were all two-sided set at α < 0.05.

The predictive performance of the model was quantified through the AUC of the ROC. Calibration of the model on the external dataset was tested using the calibration curve, and a χ^2^-test to see whether the slope and intercept are significantly different from 0 and 1, respectively. If this test is significant, it indicates the model does not fit on the external dataset. The ROC curve was plotted, and its confidence interval of 95% was calculated on 2000 stratified bootstrap replicates. In addition, the binary classification was used to create a confusion matrix, which visualizes the performance of the model by comparing the predicted BM status to the true BM status. The binary classification was performed by determining an optimal threshold on the prediction score, calculated on 2000 stratified bootstrap replicates. The metric calculated to determine the optimal cut-off was the F1-score, which takes both precision and recall into account. From this binary prediction, the sensitivity, specificity, precision, negative predictive value, accuracy, balanced accuracy, and F1-score were determined. Lastly, a two-proportion z-test was performed to determine whether there was a significant difference between the true proportions of cases in the two predicted risk groups.

The Transparent Reporting of a multivariable prediction model for Individual Prognosis or Diagnosis (TRIPOD) guidelines were adhered to.^39^ To test this adherence, the adherence form was filled in, and the TRIPOD score is reported (Supplemental Table S1). This score is a grade from 0% to 100% that gives an indication of the compliance to the TRIPOD guidelines.

## Results

### Patient inclusion

**Figure 1.**
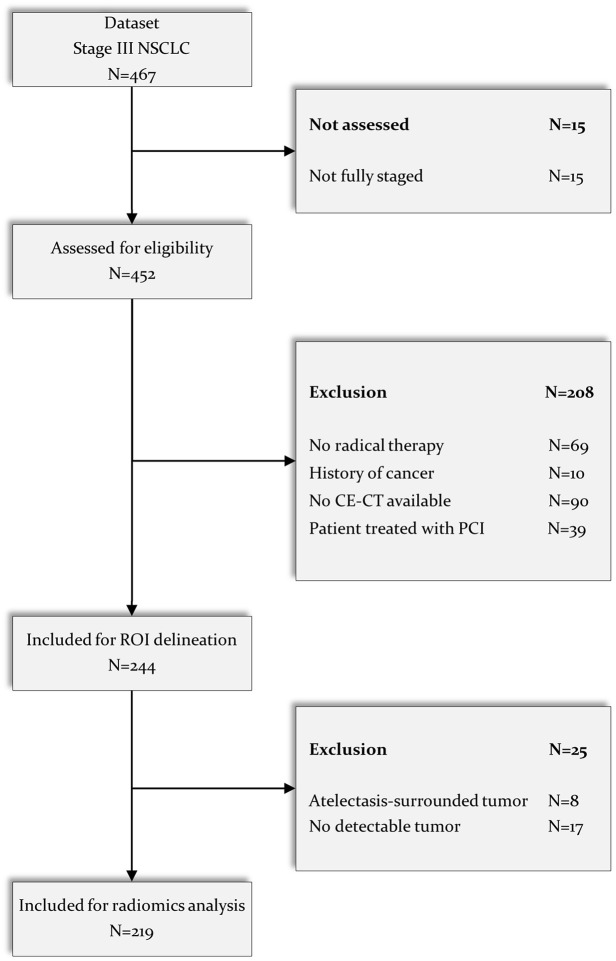
CONSORT diagram for patient selection. CE, contrast-enhanced; CT, computed tomography; MRI, magnetic resonance imaging; NSCLC, non-small-cell lung cancer; PCI, prophylactic cranial irradiation; ROI, region of interest.

A total of 467 patients with stage III NSCLC were reviewed for selection, and 248 patients were excluded for several reasons: not fully staged (*N* = 15, no adequate brain imaging, i.e. no brain MRI or dedicated brain CT as defined in the methods section); no radical therapy performed (*N* = 69); history of previous cancer (*N* = 10); no CECT of the chest available (*N* = 90); atelectasis surrounding primary tumour (*N* = 17); and no detectable primary tumour (*N* = 8). Lastly, from the NVALT-11 study, all patients with available imaging who underwent PCI were excluded (*N* = 39). As a result, 219 patients with stage III NSCLC with segmented CECT images were included for radiomics analysis. The CONSORT diagram depicting the selection process is depicted in Figure 1.

### Patient characteristics

Of the resulting 219 patients, 142 were assigned as the training dataset and 77 as the validation set. These datasets are completely independent. An overview of baseline patient characteristics is listed in Table 1. In the training set, 21 patients developed BM (incidence of 15%); in the validation dataset, 17 patients had BM development (22%). In the training dataset, 100% of the patients received a brain MRI at staging. For the validation dataset, 85.7% of the patients received an MRI, while the remaining 14.3% (11 patients) only received a CECT scan of the brain. In addition, the median FU time in the training dataset was 59.4 months [interquartile range (IQR): 40.4–71.2], and in the validation dataset 67.3 months (IQR: 42.0–83.3) (*p* = 0.05). In the entire population, patients were mostly male (61%) and mean age was 67 years at the time of NSCLC diagnosis, with 75% of patients >60 years. The majority of patients (~88%) had a WHO performance score of 0 or 1. Most patients were either current (45%) or former smokers (50%), while 3% had never smoked (2% unknown smoking status). Patients were evenly distributed in the stages IIIA and IIIB (51% and 49%, respectively), and 38% had adenocarcinoma histology. No significant differences were found in patient characteristics between the training and validation sets, except for age, where the mean age was significantly higher (*p* < 0.001) and the proportion of patients over 60 years old was significantly larger (*p* of 0.005) in the training dataset. In addition, the validation dataset received a significantly lower proportion of brain MRI (*p* < 0.001).

**Table 1.** Baseline characteristics of patients assigned to training and validation sets.

Characteristic	Training set	Validation set	Total	*p*
	*N* = 142(%)	*N* = 77(%)	*N* = 219(%)	
Gender				0.939
Male	87 (61.3)	46 (59.7)	133 (60.7)	
Female	55 (38.7)	31 (40.3)	86 (39.3)	
Age (years)				
Mean ± SD	68.6 ± 8.3	63.6 ± 8.2	66.8 ± 8.6	< 0.001
Range	47.5–88.6	47.2–85.0	47.2–88.6	
<60 years	26 (18.3)	28 (36.4)	54 (24.7)	0.005
>60 years	116 (81.7)	49 (63.6)	165 (75.3)	
WHO PS				0.293
0	53 (37.3)	26 (33.8)	79 (36.1)	
1	68 (47.9)	45 (58.4)	113 (51.6)	
2	16 (11.3)	3 (3.9)	19 (8.7)	
3	2 (1.4)	2 (2.6)	4 (1.8)	
Unknown	3 (2.1)	1 (1.3)	4 (1.8)	
Smoking status				0.163
Never	5 (3.5)	2 (2.6)	7 (3.2)	
Former	64 (45.1)	45 (58.4)	109 (49.8)	
Current	69 (48.6)	30 (39.0)	99 (45.2)	
Unknown	4 (2.8)	0 (0)	4 (1.8)	
TNM stage				0.415
IIIA	76 (53.5)	36 (46.8)	112 (51.1)	
IIIB	66 (46.5)	41 (53.2)	107 (48.9)	
Histology				0.382
Adenocarcinoma	55 (38.7)	28 (36.4)	83 (37.9)	
Squamous cell carcinoma	62 (43.7)	30 (39.0)	92 (42.0)	
Large-cell carcinoma	5 (3.5)	7 (9.1)	12 (5.5)	
Sarcomatoid	1 (0.7)	0 (0)	1 (0.5)	
LCNEC	2 (1.4)	0 (0)	2 (0.9)	
NOS	17 (12.0)	12 (15.6)	29 (13.2)	
BM diagnosed				0.241
Yes	21 (14.8)	17 (22.1)	38 (17.4)	
No	121 (85.2)	60 (77.9)	181 (82.6)	
Baseline brain MRI or brain CECT				<0.001
MRI	142 (100)	66 (85.7)	208 (95)	
Only CECT	0 (0)	11 (14.3)	11 (5)	
Treatment received				0.233
CCRT ± surgery	100 (70.4)	61 (79.2)	161 (73.5)	
SCRT ± surgery	35 (24.6)	15 (19.5)	50 (22.8)	
Radical RT	7 (4.9)	1 (1.3)	8 (3.7)	

BM, brain metastases; CCRT, concurrent chemo radiotherapy; CECT, contrast-enhanced computed tomography; LCNEC, large-cell neuroendocrine carcinoma; MRI, magnetic resonance imaging; NOS, not otherwise specified; RT, radiotherapy; SCRT, sequential chemo radiotherapy; SD, standard deviation; TNM, tumour, node, metastasis; WHO PS, World Health Organization Performance Status: 0–1: good, 2–3: poor.

### Feature selection

In total, 530 radiomics features were extracted from each CT image, and 8 clinical features were collected for each patient. After testing for univariate predictive performance and selecting features with AUC > 0.6, and excluding features with high correlation (Spearman correlation > 0.8), four relevant radiomics features (see Supplemental Section 1) and two relevant clinical features (adenocarcinoma *versus* other tumour types, and age as a continuous variable) were identified. None of the radiomics features showed high correlation (Spearman’s correlation > 0.8) with tumour volume. Table 2 shows an overview of the selected features with their respective univariate AUC, and Spearman’s correlation values with the volume.

**Table 2.** Selected clinical and radiomics features with corresponding univariate AUC, and Spearman’s correlation with volume.

Feature names		AUC	Correlation with volume
Clinical features	Adenocarcinoma *versus* other tumour type	0.66	–
	Age (continuous)	0.73	–
Radiomics features	1 mm LoG GLSZM normalized size-zone non-uniformity	0.60	−0.24
	2 mm LoG GLCM correlation	0.62	0.52
	2 mm LoG GLCM informational measure of correlation 1	0.61	−0.55
	2 mm LoG GLCM informational measure of correlation 2	0.62	0.30

AUC, area under the curve; GLCM, grey-level correlation matrix; GLSZM, grey-level size-zone matrix; LoG, Laplacian of Gaussian.

### Clinical model

**Figure 2.**
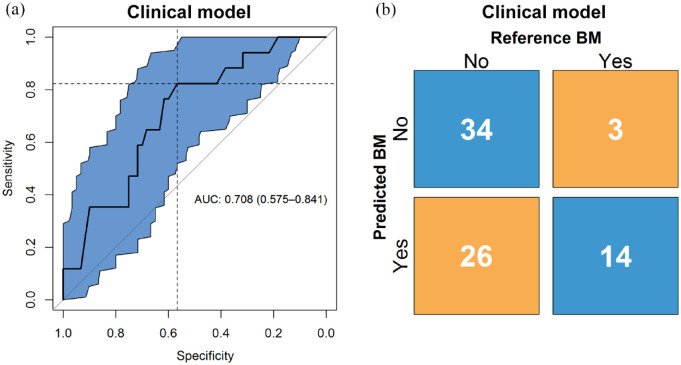
(a) ROC curve and the corresponding confidence interval of 95% in blue of the clinical model, with AUC and 95% confidence interval shown. On the *y*-axis is the sensitivity and on the *x*-axis the specificity of the model at different classification thresholds. The dashed lines show the sensitivity and specificity for the threshold that was used to make the binary prediction. (b) Confusion matrix with proportions of correct and wrong predictions made by the clinical model (*y*-axis) relative to the true labels (*x*-axis). AUC, area under the curve; ROC, receiver operating characteristic.

The performance of the predictive model built on the clinical features was evaluated in the validation set with an ROC curve, yielding an AUC of 0.71 (95% CI: 0.58–0.84), as presented in Figure 2(a). The calibration test yielded a *p* of 0.76, indicating the model fits on the external validation data. The calibration slope is found in Supplemental Figure S3. The binary prediction determined through bootstrapping gave a sensitivity and specificity of 0.82 and 0.57, respectively, which are shown in the figure represented by the dashed lines. The F1-score, the metric used to determine this cut-off, was 0.49.

The confusion matrix, shown in Figure 2(b), shows the number of correct and incorrect predictions. Of the control cases, 34 were predicted correctly; of the event cases, 14 were predicted correctly. The precision was 0.35, and the negative predictive value was 0.92. The accuracy and balanced accuracy were 0.62 and 0.70, respectively. Finally, the proportion of cases between predicted risk groups were significantly different (*p* = 0.01).

### Radiomics model

**Figure 3.**
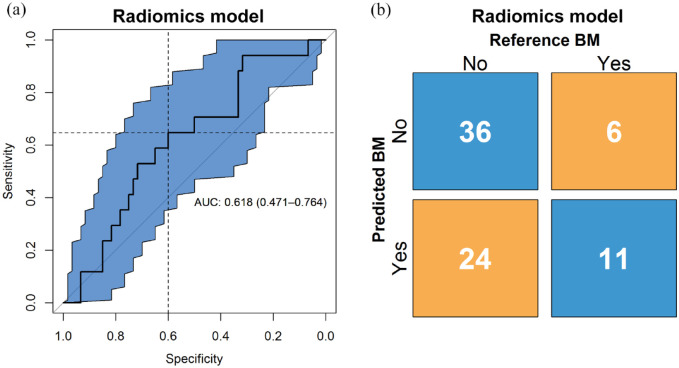
(a) ROC curve and the corresponding confidence interval of 95% in blue of the radiomics model, with AUC and 95% confidence interval shown. On the *y*-axis is the sensitivity and on the *x*-axis the specificity of the model at different classification thresholds. The dashed lines show the sensitivity and specificity for the threshold that was used to make the binary prediction. (b) Confusion matrix with proportions of correct and wrong predictions made by the radiomics model (*y*-axis) relative to the true labels (*x*-axis). AUC, area under the curve; ROC, receiver operating characteristic.

The performance of the predictive model was evaluated in the validation set with an ROC curve, yielding an AUC of 0.62 (95% CI: 0.47–0.76), as presented in Figure 3(a). The calibration test yielded a *p* < 0.001, indicating the model does not fit on the external validation data. The calibration slope is found in Supplemental Figure S4. The binary prediction determined through bootstrapping gives a sensitivity and specificity of 0.65 and 0.6, respectively, which are shown in the figure represented by the dashed lines. The F1-score, the metric used to determine this cut-off, was 0.42.

The confusion matrix, shown in Figure 3(b), shows the number of correct and incorrect predictions. Of the control cases, 36 were predicted correctly; of the event cases, 11 were predicted correctly. The precision was 0.31, and the negative predictive value was 0.86. The accuracy and balanced accuracy were 0.61 and 0.62, respectively. Finally, the proportion of cases between predicted risk groups were not significantly different (*p* = 0.13).

### Radiomics and clinical model

**Figure 4.**
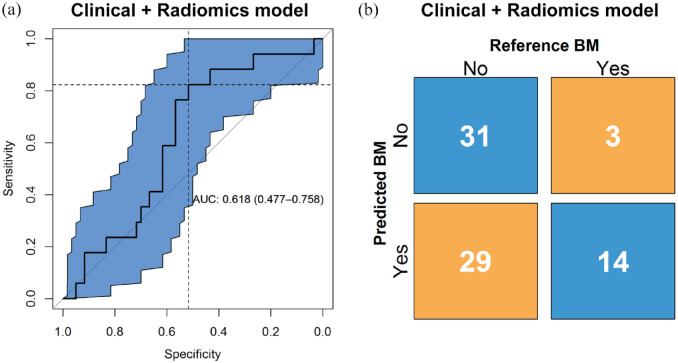
(a) ROC curve and the corresponding confidence interval of 95% in blue of the clinical and radiomics model, with AUC and 95% confidence interval shown. On the *y*-axis is the sensitivity and on the *x*-axis the specificity of the model at different classification thresholds. The dashed lines show the sensitivity and specificity for the threshold that was used to make the binary prediction. (b) Confusion matrix with proportions of correct and wrong predictions made by the clinical and radiomics model (*y*-axis) relative to the true labels (*x*-axis). AUC, area under the curve; ROC, receiver operating characteristic.

The performance of the predictive model was evaluated in the validation set with an ROC curve, yielding an AUC of 0.62 (95% CI 0.48–0.76), as presented in Figure 4(a). The calibration test yielded a *p* of 0.03, indicating the model does not fit on the external validation data. The calibration slope is found in Supplemental Figure S5. The binary prediction determined through bootstrapping gives a sensitivity and specificity of 0.82 and 0.52, respectively, which are shown in the figure represented by the dashed lines. The F1-score, the metric used to determine this cut-off, was 0.47.

The confusion matrix, shown in Figure 4(b), shows the number of correct and incorrect predictions. Of the control cases, 31 were predicted correctly; of the event cases, 14 were predicted correctly. The precision was 0.33, and the negative predictive value was 0.91. The accuracy and balanced accuracy were 0.58 and 0.67, respectively. Finally, the proportion of cases between predicted risk groups were significantly different (*p* = 0.03).

### TRIPOD statement

The TRIPOD adherence for 22 guidelines was determined, and the adherence score was calculated to be 93%. The adherence form for this study is found in Supplemental Table S1.

## Discussion

The prediction and prevention of BM development in patients with radically treated stage III NSCLC is a major issue, as BM has a detrimental effect on survival and QoL.^10,11^ Preventive strategies such as PCI exist, but come at a cost of neurocognitive decline, and PCI has been shown to not be associated with an OS benefit in patients with stage III NSCLC not selected for BM risk.^4^ Therefore, future studies evaluating new preventive treatments or the effects of regular screening should focus on those at high risk of BM. Patients with a low risk of BM could be spared PCI or intense imaging FU. This strategy requires a model that accurately separates high-risk from low-risk stage III NSCLC patients.

In this multicentre study, we developed a radiomics model based on four radiomics features extracted from the primary lung tumour on CECT imaging and combined this with existing clinical predictors of BM. The first feature is based on a GLSZM matrix, which quantifies the number and size of homogeneous intensity patches found within the ROI. The normalized size-zone non-uniformity feature based on this matrix measures variability of these size zones, with a higher score meaning less homogeneous areas with the same intensity present in the ROI, that is, more heterogeneity. The remaining three features are based on a GLCM matrix, which measures the frequency in which certain combinations of pixel intensity values are found. The features correlation, Informational Measure of Correlation 1 (IMC1), and IMC2 based on this matrix all measure whether correlations between certain intensity values can be found within the ROI. A higher value would mean that more homogeneous areas exist within the ROI, while a lower value means the intensity values are more randomly spread throughout the ROI, which is again a measure of heterogeneity.

We found that in a patient population of 219 (training *N* = 142 and validation *N* = 77), the addition of radiomics was not able to improve the predictive performance of a model based solely on clinical factors. This result may indicate that, for the aforementioned population size, factors other than phenotypical characteristics of the tumour are more important in the incidence of BM, such as histology and age, as shown in the features selected for the clinical model.

To our knowledge, few studies have been undertaken on the topic of BM prediction using a combination of clinical and radiomics features. We found three radiomics studies with a comparable study design, shown contrasted to our study in Table 3.^29–31^ While one of the radiomics models has significantly higher performance (AUC of 0.85 *versus* 0.62), these studies shared a low number of patients as well as BM events, a lack of external validation, and a lack of full staging compared to the current study, resulting in low reliability of the results.

**Table 3.** Study parameters of radiomics studies on BM or DM prediction in NSCLC.

Study name	Coroller *et al.*^29^	Chen *et al.*^30^	Xu *et al.*^31^	Present study (2021)
Study population	Stage II–III/adenocarcinoma	T1-stage/adenocarcinoma	Stage III–IV/ALK positive	Stage IIIA/B
Sample size	*N* = 182	*N* = 89	*N* = 105	*N* = 219
Primary outcome	DM	BM	BM	BM
Number of events in FU	69 (37.9%)	35 (39.3%)	27 (25.7%)	38 (17.4%)
Staging	?	T1/N-stage based on non-CECT	‘By medical images’	Full imaging
^18^F-FDG-PET-CT	−	−	?	+
Brain MRI/CECT (% MRI received)	(N/A)	+ (Not reported)	+ (Not reported)	+ (95)
Chest CECT	−	−	+	+
Pathological analysis	Pathologically confirmed lung adenocarcinoma	‘Pathologically confirmed disease’	Pathologically confirmed ALK	–
Imaging modality	Planning CT + GTV (patients excluded if CTx/surgery was before RTx scheduled date)	Pre-treatment non-CECT	Pre-treatment CECT + RTstruct	Pre-treatment CECT + RTstruct
Predictive performance (95% CI)	CI > 0.6 (−)	AUC 0.85 (0.767–0.933)	AUC 0.64 (0.501–0.783)	AUC 0.62 (0.47–0.76)
Strengths	(+) Pathologically confirmed (+) Pre-treatment CT	(+) Pathologically confirmed (+) BM exclusion at baseline (+) Pre-treatment CT	(+) Pathologically confirmed (+) BM exclusion at baseline (+) Diagnostic chest CECT/pre-treatment	(+) Pathologically confirmed (+) BM exclusion at baseline (+) Diagnostic chest CECT/pre-treatment (+) External validation
Limitations	(−) Unclear staging (−) Small sample size (−) GTV not specified (LN included?) (−) DM locations not specified (−) Planning CT	(−) Unclear staging; T1/N-stage determined with non-CECT (−) Small sample size	(−) Unclear staging; PET-CT not reported (−) Small sample size (−) GTV not specified (LN included?) (−) Relatively low number of BM	(−) Relatively low number of BM

ALK, anaplastic lymphoma kinase; BM, brain metastasis; (CE-)CT, contrast-enhanced computed tomography; CTx, chemotherapy; DM, distant metastasis; ^18^F-FDG-PET-CT, ^18^F-fluorodeoxyglucose positron emission tomography-computed tomography; FU, follow-up; GTV: gross tumor volume LN, lymph node; MRI, magnetic resonance imaging; N, lymph node stage; NSCLC, non-small-cell lung cancer; RTx, radiotherapy; T1, tumour stage 1.

Data quality should be a priority when selecting the study population.^40^ Especially, the large disease heterogeneity in stage III NSCLC emphasizes the importance of correct staging with the appropriate imaging modalities, as disease stage directly influences treatment options and prognosis.^5^ For the previously reported studies, either ^18^F-FDG-PET-CT or dedicated brain imaging (brain MRI or dedicated brain CT) was not mandatory, while in the present study only adequately staged patients were included for analysis. Therefore, in the previously reported studies, patients with occult BM could have been enrolled. For example, 15–21% of patients with stage III NSCLC have asymptomatic BM and without dedicated imaging, these will be missed.^41,42^ Asymptomatic BM are diagnosed on MRI in approximately 5% of patients that underwent a dedicated brain CT (with contrast and the correct field of view), and in 16% of patients that underwent an ^18^F-FDG-PET-CT with a low-dose CT of the brain.^34,42^ All patients in our study received dedicated brain imaging, with 95% MRI and 5% CECT. Therefore, risk of bias due to undetected baseline BM is low in our study.

A further point of strength of this study is the use of ^18^F-FDG-PET-CT alongside CECT images during contouring. In the field of radiation therapy, the differentiation of lung tumour from post-obstructive atelectasis is a well-recognized problem, which even contrast enhancement cannot always resolve. As ^18^F-FDG-PET-CT has proven utility during tumour delineation for radiation planning purposes, this may have significantly increased the delineation accuracy of the CECT images in our study.^43^

There may be a number of different reasons why the radiomics model failed to accurately predict patients at risk for BM. This study primarily focused on the selection of CECT images in consideration of delineation accuracy, as CECT is more specific in differentiating different tissue types, especially in case of mediastinal invasion, which often occurs in stage III NSCLC.^44^ However, this may have diminished the discriminatory performance of the model, since recent studies have found differences between CECT and non-CECT radiomics features.^45,46^ In addition, CECT was associated with variability of radiomics features due to differences in contrast uptake; a concept which is strongly influenced by patient variables which impact contrast distribution, for example, age and weight.^47^ Given that patient-related factors are a permanent source of variability (with any imaging modality), efforts should be directed at homogenizing datasets in terms of contrast enhancement and investigating CECT robust features. Furthermore, despite the strict selection of CECT with the same reconstruction protocol and slice spacing, there were still differences in imaging parameters and the images were not fully standardized. The collected images were not standardized to one acquisition and reconstruction protocol before or during the studies. Furthermore, due to the retrospective nature of the study, we were not able to perform phantom scans on the different scanners. Performing phantom studies or applying a different harmonization method is likely needed to harmonize images and make reproducible models. This should be standard practice in a radiomics protocol.^48–50^

This study was performed on a homogeneous patient group regarding stage, only including stage IIIA and IIIB tumours. However, stage III NSCLC is known for its heterogeneity regarding varying tumour sizes and the pattern of lymph node metastasis (e.g. a T1N3 *versus* a T4N0 tumour).^51^ This could further explain the inability of the model to predict BM, and while it was not in the scope of the current study due to a lack of data in the NCT01282437 study, investigating further clinical features that describe the risk of high T-status *versus* high N-status, or total tumour volume could be investigated, as Won *et al.*^17^ have shown these features have predictive power. The clinical features selected, age and histology, are not directly affected by this shortcoming. Although selection based on stage may increase homogeneity, it could also overlook the complexity of BM risk. For instance, primary tumour size alone is inadequate in predicting disseminating tumour behaviour, that is, small tumours with extensive N-status have previously been described to metastasize early, whereas large tumours with limited N-stage may not at all.^52^ Therefore, a critical evaluation of the target population and the associated clinical implications is necessary in conducting relevant research.

Compared to previous studies that report a BM incidence of approximately 30%, the incidences of BM in the training and validation set were significantly lower at 15% and 22%, respectively.^4^ Both NVALT11 and NL3335 had a median FU time largely exceeding 2 years, while most BM occur within 2 years of the initial staging of NSCLC.^33^ Therefore, inadequate FU time is not an explanation. For NVALT11 (control arm 28% BM in FU), not all scans could be retrieved, and indeed more scans were retrieved from patients without BM. In addition, almost all patients included had a baseline brain MRI and not only a CECT. It is known that MRI is slightly superior (in 5% of patients additional BM detected after negative CECT) in detecting asymptomatic BM in stage III NSCLC and this also could have resulted in a lower BM incidence in the FU.^34^

The small sample size, even though larger datasets were used compared to previous studies, and different imaging parameters are both well-known sources of variability in radiomics that limit reproducibility.^53^ Furthermore, manual tumour delineations are prone to inter-observer variability, which affect the stability of radiomics features.^54^ Taken together, these aspects may explain the limited performance of the radiomics model and require further attention. Therefore, our future work will address these limitations by optimizing the radiomics model through expanding the sample size and reducing data heterogeneity, using imaging phantoms and standardization methods in the radiomics pipeline, and through image and feature harmonization. While clinical factors seem to outperform radiomics features, with the current sample size the results are inconclusive with regard to the complementary predictive role of CT-based radiomics.

Future radiomics studies could also focus on utilizing the additional imaging performed during the standard diagnostic workup of patients with stage III NSCLC. These imaging modalities, for example, dedicated brain MRI or CECT together with ^18^F-FDG-PET-CT, may have additional value in BM prediction. For instance, brain MRI features might reveal micro metastases indiscernible to the human eye, and may aid in the early detection, whereas tumour heterogeneity captured by ^18^F-FDG-PET-CT uptake pattern may further characterize tumour aggressiveness.^55^ Accordingly, imaging modality-specific features could be integrated to form a robust radiomics signature.

Finally, other artificial intelligence approaches, such as deep learning models, have shown to be able to perform risk prediction on clinical images.^56^ While these methods usually require larger datasets to achieve significant results, they should be investigated in future studies for their complementary value in predicting the risk of BM. Other machine learning methods such as recursive feature elimination or least absolute shrinkage and selection operator to select features exist, which have shown to be able to improve performance of predictive models. However, with the current study setup and study population size, the feature selection through univariate predictive performance was found to achieve the highest performance.

## Conclusion

A model based on known clinical predictors of BM development (age and tumour histology) is able to predict BM development in patients with radically treated stage III NSCLC with moderate precision, with an AUC of 0.71 (model available on www.ai4cancer.ai). This model did not improve with the addition of CT-based radiomics features. Future work will focus on optimizing the radiomics model by expanding the dataset, investigating more clinical features, other imaging modalities, data harmonization, and reducing data heterogeneity.

## Supplemental Material

sj-docx-1-tam-10.1177_17588359221116605 – Supplemental material for Investigation of the added value of CT-based radiomics in predicting the development of brain metastases in patients with radically treated stage III NSCLC Click here for additional data file. Supplemental material, sj-docx-1-tam-10.1177_17588359221116605 for Investigation of the added value of CT-based radiomics in predicting the development of brain metastases in patients with radically treated stage III NSCLC by Simon A. Keek, Esma Kayan, Avishek Chatterjee, José S. A. Belderbos, Gerben Bootsma, Ben van den Borne, Anne-Marie C. Dingemans, Hester A. Gietema, Harry J. M. Groen, Judith Herder, Cordula Pitz, John Praag, Dirk De Ruysscher, Janna Schoenmaekers, Hans J. M. Smit, Jos Stigt, Marcel Westenend, Haiyan Zeng, Henry C. Woodruff, Philippe Lambin and Lizza Hendriks in Therapeutic Advances in Medical Oncology

## Declarations

**Ethics approval and consent to participate:** The collection of the imaging data for the current study was approved by the Medical Ethics Review Committee of Maastricht UMC+ (2017-0317), and, if applicable, by institutional review boards of the other participating centres. The ethics committee approved the waiver of informed consent.

**Consent for publication:** Not applicable.

**Availability of data and materials:** Not applicable.
